# Reinforce, Don't Sacrifice: A Dentin-Preserving Alternative to Post-and-Core Restorations Using Ribbond, everStickPOST, and Interlig Fibers

**DOI:** 10.7759/cureus.116200

**Published:** 2026-09-13

**Authors:** Sanjeev Srivastava, Prachi Jha, Shubhangi Shubhangi, Alisha Roy, Aparna Singh

**Affiliations:** 1 Conservative Dentistry and Endodontics, Sardar Patel Post Graduate Institute of Dental and Medical Sciences, Lucknow, IND

**Keywords:** adhesive bonding, fracture resistance, glass fibers, minimally invasive endodontics, polyethylene fibers

## Abstract

Restoration of structurally compromised endodontically treated anterior teeth remains challenging because additional radicular dentin removal during post-space preparation may further compromise the remaining tooth structure. Fiber-reinforced systems provide a conservative approach by permitting intracanal reinforcement while minimizing additional removal of radicular dentin. This three-case clinical report describes the rehabilitation of three structurally compromised, endodontically treated maxillary central incisors using three different fiber-reinforced systems: everStickPOST, Ribbond, and Interlig. In all three cases, treatment emphasized preservation of the remaining radicular and pericervical dentin. In the everStickPOST case, 5 mm of apical gutta-percha was preserved, whereas approximately 4 mm of coronal gutta-percha was removed in the Ribbond and the Interlig cases to preserve pericervical dentin. The fibers were individually adapted to the prepared canal anatomy and incorporated into adhesive composite core restorations. In the Interlig case, bioactive glass treatment followed by riboflavin and 0.2% chitosan pretreatment was additionally performed before fiber placement. A full-coverage crown was provided in the everStickPOST and Ribbond cases because of the extent of coronal structural loss, whereas the Interlig case was restored with direct composite restorations because sufficient coronal and pericervical tooth structure remained. All three cases demonstrated satisfactory clinical and radiographic outcomes at the reported clinical evaluation. These cases illustrate the potential application of customized fiber-reinforced intracanal reinforcement as a conservative strategy for restoring structurally compromised endodontically treated anterior teeth. The treatment approach emphasizes preservation of radicular dentin, individualized adaptation to canal anatomy, and selection of the definitive coronal restoration according to the residual tooth structure and biomechanical requirements.

## Introduction

Loss of coronal tooth structure secondary to caries, trauma, and repeated restorative procedures, together with removal of pericervical dentin during endodontic access and canal preparation, can substantially reduce the structural integrity and fracture resistance of endodontically treated teeth. Excessive enlargement and tapering of the root canal may further compromise the remaining radicular dentin and increase susceptibility to fracture under functional loading [1].

Historically, cast metal post-and-core systems were widely used for the restoration of extensively compromised endodontically treated teeth because of their rigidity and ability to provide retention for definitive full-coverage restorations. However, the substantially higher elastic modulus of metallic posts compared with dentin may result in unfavorable stress distribution at the post-dentin interface. Concentration of stresses within the root may contribute to catastrophic fracture patterns, including vertical root fracture, which can render the tooth unrestorable [2,3].

The development of prefabricated and customized fiber-reinforced systems has facilitated a more conservative approach to the restoration of endodontically treated teeth. Fiber-reinforced composite (FRC) systems, including polyethylene and glass fiber systems, possess mechanical characteristics that more closely approximate those of dentin than conventional metallic posts. Their use may facilitate more favorable stress distribution while reducing the need for extensive removal of radicular dentin [4-6].

In addition to their biomechanical advantages, fiber-reinforced systems offer favorable esthetic characteristics because of their translucency and compatibility with adhesive restorative procedures. These properties are particularly advantageous in the anterior region, where preservation of natural tooth structure and optical integration are important treatment considerations.

Several fiber-reinforced systems are available for clinical use, with differences in fiber chemistry, architecture, surface treatment, matrix composition, and handling characteristics. Ribbond (Ribbond Inc., Seattle, WA) consists of ultra-high-molecular-weight polyethylene (UHMWPE) fibers arranged in a leno-weave architecture. The fibers undergo gas-plasma surface treatment, which increases surface energy and improves wettability, facilitating resin infiltration and interfacial bonding [7].

everStickPOST (GC Corporation, Tokyo, Japan) is a pre-impregnated, unidirectional E-glass fiber system incorporated within a semi-interpenetrating polymer network matrix. Its partially polymerized matrix allows the material to remain moldable during clinical manipulation and permits adaptation to the individual root canal or coronal defect before final polymerization.

Interlig (Angelus, Londrina, Brazil) is a braided E-glass fiber ribbon that has been used for periodontal splinting and reinforcement of composite restorations and post-core structures. Its fiber architecture and handling characteristics differ from those of both woven polyethylene and unidirectional glass fiber systems.

The present case series describes the clinical management of three structurally compromised, endodontically treated anterior teeth using three different fiber-reinforced systems: everStickPOST, Ribbond, and Interlig. The treatment approach emphasized preservation of the remaining radicular dentin and individualized selection of definitive coronal restoration according to the amount and quality of residual tooth structure.

## Case presentation

Case 1

A 21-year-old female patient reported to the Department of Conservative Dentistry and Endodontics with a dislodged coronal restoration involving the maxillary left central incisor (#21) (Figure 1, Panel A). The patient reported loss of the restoration during function and denied spontaneous pain, thermal sensitivity, or swelling. Clinical examination revealed extensive loss of coronal tooth structure, with less than 50% of the original coronal structure remaining. The remaining tooth structure was considered adequate for conservative fiber-reinforced rehabilitation. The tooth was non-tender to percussion and palpation, with no sinus tract, localized swelling, or abnormal mobility. Periodontal examination revealed probing depths of 2-3 mm circumferentially, with no localized periodontal pocketing or bleeding on probing.

**Figure 1:**
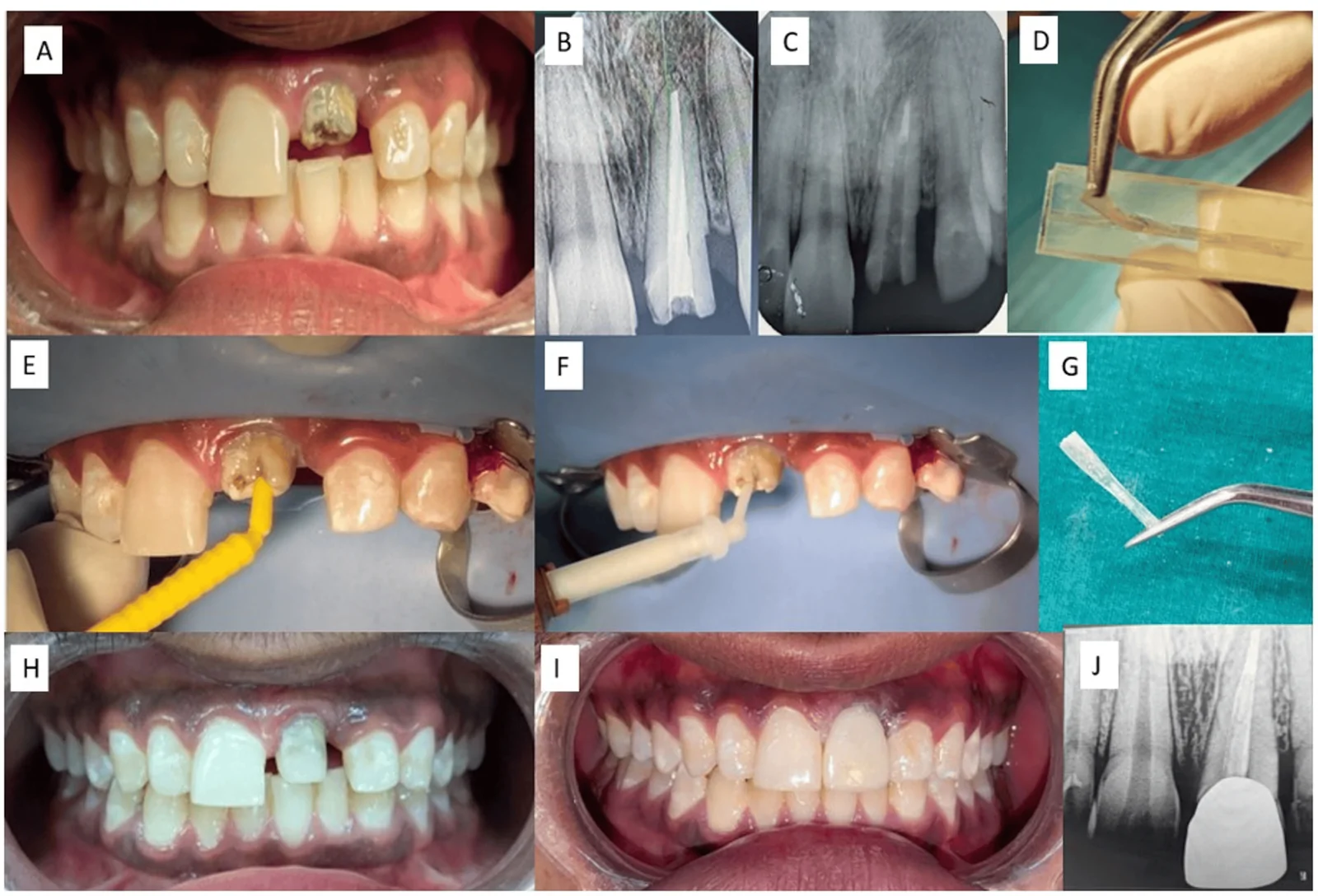
Clinical and radiographic sequence of the everStickPOST fiber-reinforced restoration in Case 1 (A) Preoperative intraoral photograph showing extensive loss of coronal tooth structure; (B) preoperative periapical radiograph; (C) periapical radiograph following removal of the coronal gutta-percha; (D) everStickPOST fiber separated from the silicone sheath; (E) application of the adhesive system within the prepared canal; (F) placement of dual-cure resin cement into the canal; (G) everStickPOST fiber adapted in a fanned configuration for intracanal reinforcement and core retention; (H) composite core build-up following fiber placement; (I) postoperative intraoral photograph following definitive full-coverage crown placement; and (J) postoperative periapical radiograph demonstrating the completed restoration.

Preoperative periapical radiographic examination demonstrated a previously root canal-treated maxillary left central incisor with radiopaque root canal filling material extending to the radiographic apex and no evident periapical radiolucency (Figure 1, Panel B). Based on the clinical and radiographic findings, the tooth was classified as previously treated with normal apical tissues and was deemed suitable for conservative post-endodontic rehabilitation. After discussion of the treatment options, informed consent was obtained for fiber-reinforced intracanal restoration followed by definitive full-coverage rehabilitation.

The procedure was performed under rubber dam isolation. A sequential drilling of a Peeso Reamer was used to remove the coronal portion of gutta-percha while preserving 5 mm of apical gutta-percha to maintain an adequate apical seal. A periapical radiograph was obtained following gutta-percha removal to verify the prepared canal space and the remaining apical filling (Figure 1, Panel C). The canal was subsequently irrigated with saline and dried with paper points.

The required length of the everStickPOST was determined by measuring the prepared intracanal space and allowing approximately 4 mm for coronal extension into the composite core. A 1.2-mm-diameter everStickPOST was cut to the required length and separated from its silicone sheath using sterile tweezers (Figure 1, Panel D). The post was trial-inserted to verify its length and adaptation within the prepared canal.

The prepared canal walls were conditioned using the adhesive system selected for post-cementation (Figure 1, Panel E). The prepared canal space was subsequently filled with dual-curing resin cement (ParaCore; Coltène, Altstätten, Switzerland) (Figure 1, Panel F). A pre-fitted hand plugger was used to adapt the apex and facilitate intimate adaptation to the prepared canal walls.

Before cementation, the everStickPOST surface was activated with the appropriate light-curable, unfilled, solvent-free methacrylate resin according to the manufacturer's instructions. The post was then shaped into a U-shaped configuration, with the coronal portion fanned to increase the surface area available for incorporation into the composite core (Figure 1, Panel G). The customized post was inserted into the resin-filled canal and adapted apically using a pre-fitted hand plugger. Polymerization was subsequently performed according to the manufacturer's recommended curing protocol. Following post placement, the coronal extension of the fiber was incorporated into the composite core build-up (Figure 1, Panel H).

The remaining coronal tooth structure was etched using 34% phosphoric acid etchant (Tooth Conditioner Gel Syringe, Dentsply), followed by application of Prime & Bond NT (Dentsply DeTrey GmbH, Konstanz, Germany). Filtek Z250 composite resin (3M ESPE, St. Paul, MN) was subsequently placed incrementally around the coronal extension of the fiber to complete the reinforced composite core. The composite core was subsequently contoured and finished before definitive prosthetic rehabilitation.

Because of the extensive loss of coronal tooth structure, a full-coverage crown was selected as the definitive restoration to provide extracoronal reinforcement and functional rehabilitation (Figure 1, Panel I). A postoperative periapical radiograph was obtained to document the completed restoration and intracanal fiber-reinforced reconstruction (Figure 1, Panel J).

At a 12-month clinical follow-up, the patient remained asymptomatic, and the restored tooth was functional without tenderness to percussion or palpation, abnormal mobility, or localized soft-tissue pathology. Periodontal probing depths remained within the 2-3 mm range, without localized periodontal pocketing or bleeding on probing. The definitive crown demonstrated satisfactory clinical adaptation, contour, and esthetic integration. The 12-month periapical radiograph demonstrated no evidence of a newly developed periapical radiolucency or other radiographic periapical complication. The overall clinical and radiographic outcome was considered satisfactory.

Case 2

A 28-year-old male patient presented with a fractured maxillary left central incisor (#21), associated with substantial loss of coronal tooth structure (Figure 2, Panel A). The patient reported fracture of the previously restored tooth during function, with no history of spontaneous pain or thermal sensitivity. Clinical examination revealed a structurally compromised but restorable maxillary left central incisor. The tooth was non-tender to percussion and palpation, with no sinus tract, swelling, or abnormal tooth mobility. Periodontal examination revealed probing depths of 2-3 mm circumferentially, with no bleeding on probing or localized periodontal pocketing. The tooth was considered suitable for conservative adhesive rehabilitation.

**Figure 2:**
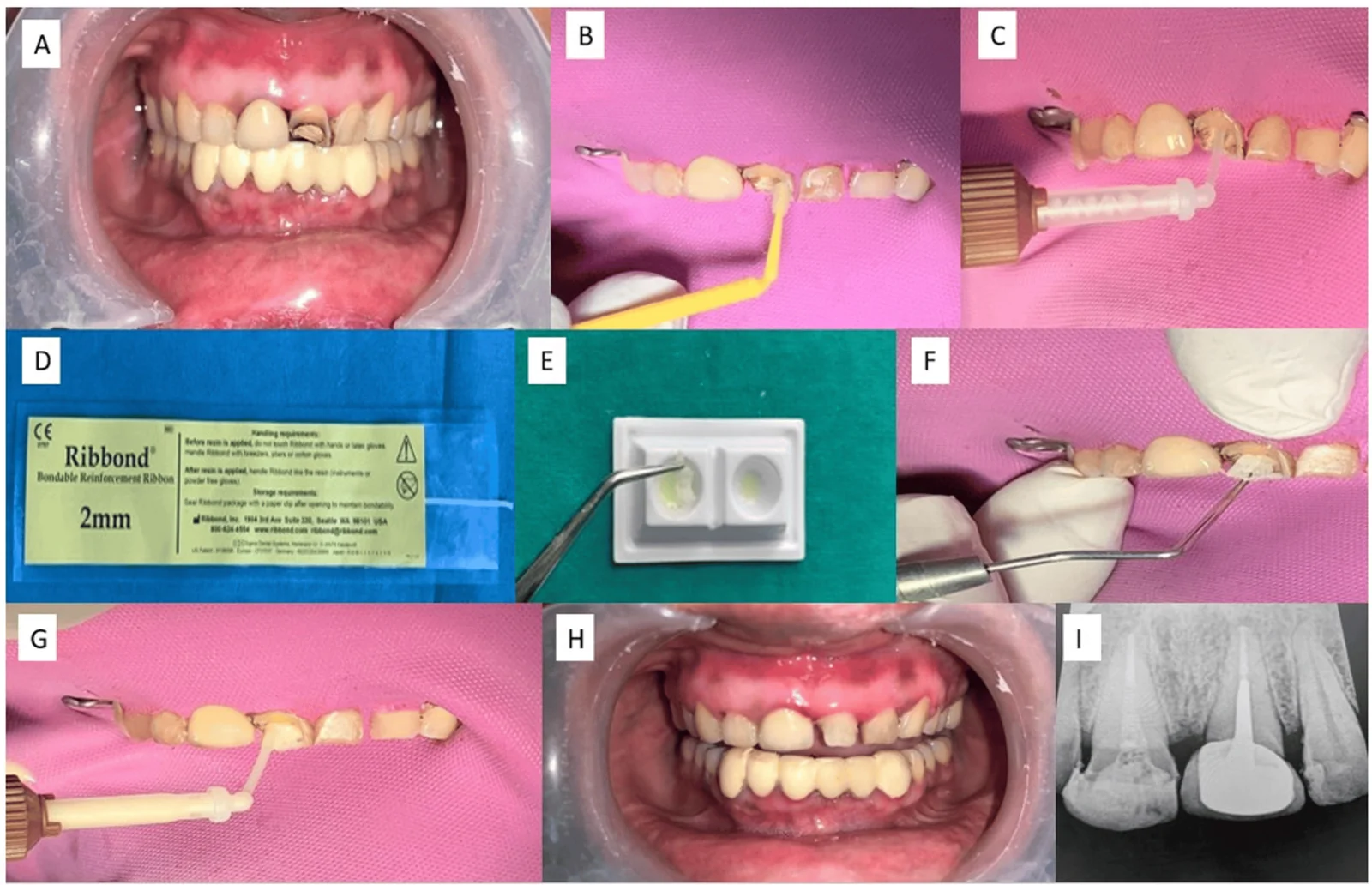
Clinical sequence of Ribbond fiber-reinforced restoration in Case 2 (A) Preoperative intraoral photograph showing the fractured maxillary left central incisor; (B) application of the bonding agent; (C) placement of dual-cure resin cement into the prepared canal; (D) 2-mm Ribbond fiber; (E) Ribbond fiber wetted with silane coupling agent; (F) insertion of the Ribbond fiber into the root canal; (G) composite core build-up following fiber placement; (H) tooth preparation for definitive placement of crown; and (I) postoperative periapical radiograph demonstrating the completed restoration.

Preoperative periapical radiographic examination demonstrated a previously root canal-treated maxillary left central incisor with satisfactory obturation and no evident periapical radiolucency. Based on the clinical and radiographic findings, the tooth was classified as previously treated with normal apical tissues. Because sufficient coronal and radicular tooth structure remained for conservative rehabilitation, intracanal fiber reinforcement was planned to reinforce the residual tooth structure while minimizing additional removal of radicular dentin.

Under rubber dam isolation, approximately 4 mm of coronal gutta-percha was removed from the root canal to create a limited intracanal space for fiber placement. The preparation was intentionally restricted to the coronal portion of the canal to preserve the remaining radicular dentin. The prepared canal walls were subsequently conditioned with the bonding agent (Figure 2, Panel B), followed by placement of dual-cure resin cement into the prepared canal space (Figure 2, Panel C).

A 2-mm-wide polyethylene fiber ribbon (Ribbond) was selected according to the dimensions of the prepared canal (Figure 2, Panel D). The fiber was wetted with a silane coupling agent to facilitate interaction with the resin matrix (Figure 2, Panel E). The treated fiber was subsequently inserted into the prepared root canal and adapted closely against the canal walls (Figure 2, Panel F). Additional resin material was applied to facilitate intimate adaptation and incorporation of the fiber within the canal space. Care was taken to maintain the conservative nature of the preparation and avoid unnecessary enlargement of the root canal.

Following intracanal fiber placement and adaptation, adhesive composite resin core build-up was completed by incorporating the coronal extension of the fiber into the composite restorative material. The remaining coronal tooth structure was reconstructed incrementally, followed by anatomical contouring, finishing, and polishing. In view of the adequate residual coronal and pericervical tooth structure, a direct composite restoration was done, with placement of a full-coverage crown (Figure 2, Panels G-H).

The completed restoration was evaluated clinically and radiographically. At a 12-month follow-up, the tooth remained asymptomatic and functional, with no tenderness to percussion or palpation, no mobility, no localized periodontal pocketing, and no evidence of restoration failure. Periodontal probing depths remained within the 2-3 mm range, and the restoration demonstrated satisfactory marginal adaptation and esthetic integration. The 12-month postoperative periapical radiograph demonstrated maintenance of the periapical status without development of a new periapical radiolucency (Figure 2, Panel I). Overall, the case demonstrated satisfactory short-term clinical and radiographic performance of a conservative Ribbond fiber-reinforced direct composite restoration.

Case 3

A 26-year-old female patient presented with a crown fracture involving the maxillary right central incisor (#11) (Figure 3, Panel A). The patient reported fracture of the anterior tooth during mastication, with no history of spontaneous pain, thermal sensitivity, or swelling. Clinical examination revealed a substantial loss of coronal tooth structure involving the maxillary right central incisor, while adequate coronal and pericervical tooth structure remained for conservative adhesive rehabilitation. The tooth was non-tender to percussion and palpation, with no sinus tract, swelling, or abnormal mobility. Periodontal examination revealed probing depths of 2-3 mm circumferentially, with no localized periodontal pocketing or bleeding on probing. The tooth was considered restorable, and a conservative fiber-reinforced adhesive restoration was planned.

**Figure 3:**
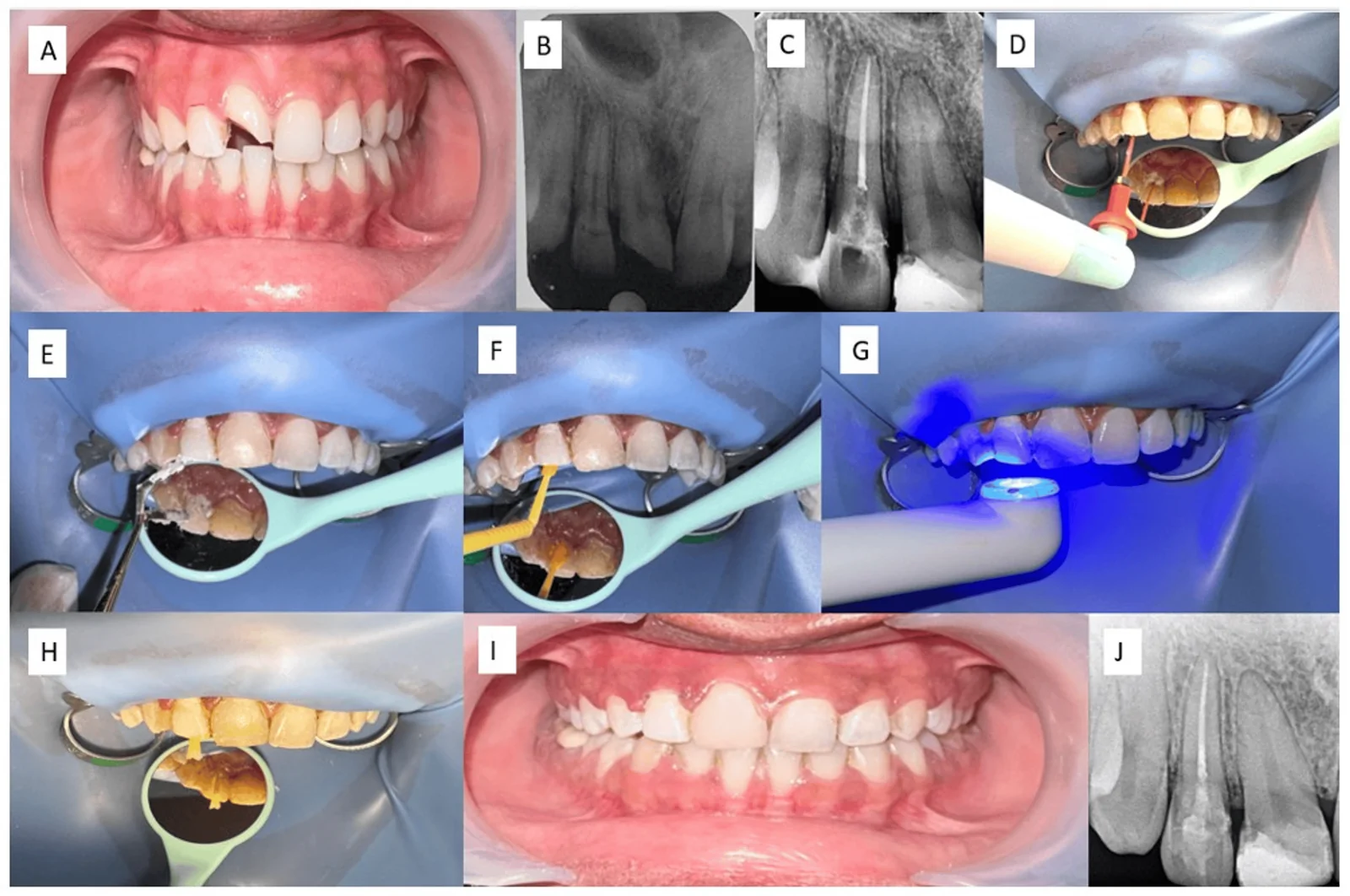
Clinical and radiographic sequence of Interlig fiber-reinforced restoration in Case 3 (A) Preoperative intraoral photograph showing the fractured maxillary right central incisor; (B) preoperative periapical radiograph; (C) periapical radiograph following removal of the coronal gutta-percha; (D-E) bioactive glass activation within the root canal; (F-G) application of the pretreatment agents followed by photoactivation; (H) fanning and adaptation of the Interlig fiber; (I) postoperative intraoral photograph; and (J) postoperative periapical radiograph demonstrating the completed restoration.

A preoperative periapical radiograph of the maxillary right central incisor was obtained, and root canal treatment (RCT) was subsequently performed before proceeding with the fiber-reinforced restoration (Figure 3, Panel B). Because the remaining coronal and pericervical tooth structure was favorable, a dentin-preserving intracanal reinforcement protocol followed by direct composite restoration was selected.

Under rubber dam isolation, gutta-percha was removed 4 mm coronally. The preparation was intentionally limited to the coronal portion of the canal to minimize further removal of radicular dentin. A periapical radiograph was obtained to verify the extent of coronal gutta-percha removal and the prepared canal space (Figure 3, Panel C).

As an adjunctive dentin-biomodification procedure, bioactive glass powder was mixed with distilled water at a 1:5 ratio and introduced into the prepared canal. The bioactive glass was activated using a sonic activator, with the tip positioned 2 mm short of the working length (Figure 3, Panel D). Three activation cycles of 20 seconds each were performed, with the activation sequence documented (Figure 3, Panel E). The biomodification protocol was intended to enhance interaction between the restorative complex and the remaining dentin while maintaining the conservative nature of the canal preparation.

Following bioactive-glass treatment, the canal walls were pretreated with riboflavin combined with 0.2% chitosan. The pretreatment solution was applied using a micro-applicator tip (Figure 3, Panel F) and subsequently photoactivated for 120 seconds (Figure 3, Panel G). This adjunctive protocol was incorporated before placement of the intracanal fiber reinforcement.

An Interlig fiber ribbon was subsequently adapted within the prepared canal in a fanned configuration to provide intracanal reinforcement (Figure 3, Panel H). The fiber was positioned to maximize adaptation to the prepared canal while reinforcing the residual radicular and coronal tooth structure. Composite resin core build-up was subsequently performed, incorporating the fiber into the adhesive restorative complex.

As sufficient sound coronal and pericervical tooth structure remained and the restoration could be supported by the residual tooth structure, a full-coverage crown was not selected. The tooth was therefore restored definitively with a direct composite resin restoration. The restoration was anatomically contoured, finished, and polished. The postoperative intraoral appearance demonstrated satisfactory reconstruction of the fractured anterior tooth (Figure 3, Panel I). A postoperative periapical radiograph was subsequently obtained to evaluate the completed intracanal reinforcement and coronal restoration (Figure 3, Panel J).

At 12 months of clinical follow-up, the patient remained asymptomatic, and the restored tooth was functional without tenderness to percussion or palpation, abnormal mobility, or soft-tissue pathology. Periodontal probing depths remained within the 2-3 mm range, without localized periodontal pocketing or bleeding on probing. Clinically, the direct composite restoration demonstrated satisfactory marginal adaptation, contour, and esthetic integration. The 12-month periapical radiograph demonstrated no development of a new periapical radiolucency or other radiographic evidence of treatment-related complications. The overall clinical and radiographic outcome was considered satisfactory.

## Discussion

The restoration of endodontically treated teeth is strongly influenced by the amount, distribution, and quality of the remaining tooth structure, while the use of an intracanal post represents only one component of the overall restorative strategy. Additional dentin removal during post-space preparation may further compromise structurally weakened teeth, particularly when the remaining radicular and pericervical dentin is limited. Consequently, contemporary restorative approaches emphasize preservation of sound tooth structure and conservative adhesive reinforcement rather than unnecessary enlargement of the root canal solely to accommodate a rigid post [8-9]. However, post-placement should not be considered universally unnecessary, as clinical evidence suggests that appropriately selected endodontically treated teeth may benefit from intracanal retention when the remaining coronal structure is insufficient for adequate core retention.

Cast metal post-and-core systems have traditionally been used because of their rigidity and ability to provide reliable core retention. However, their substantially higher elastic modulus relative to dentin may alter stress distribution within the restored root. Under unfavorable geometric and loading conditions, this difference in stiffness may contribute to localized stress concentrations at the post-dentin interface and in regions of reduced dentinal thickness, potentially increasing the risk of unfavorable root fracture [8]. The clinical behavior of a post-restored tooth, however, is multifactorial and depends on the amount of residual tooth structure, ferrule, post geometry, cementation procedure, coronal restoration, and functional loading rather than on post rigidity alone.

Preparation for cast and prefabricated rigid posts may require removal of additional radicular dentin to create adequate post-space dimensions. This consideration is particularly important in teeth with thin roots, reduced pericervical dentin, or irregular canal morphology, where unnecessary enlargement may compromise structural integrity [9]. Prefabricated rigid posts may also provide less intimate adaptation in irregular or anatomically variable canals, potentially necessitating further dentin removal or resulting in a relatively thick and nonuniform luting layer [10]. These considerations support the use of restorative strategies that maximize preservation of the remaining dentin while providing sufficient retention and reinforcement for the planned coronal restoration.

Fiber-reinforced post systems have consequently gained considerable clinical application because their elastic modulus is generally closer to that of dentin than that of metallic posts. This mechanical compatibility may contribute to a more favorable distribution of stresses within the restored tooth and may reduce the likelihood of catastrophic root fracture under certain loading conditions [11]. Fiber-reinforced restorations have also been associated with a greater proportion of repairable or non-catastrophic failure patterns. Nevertheless, the clinical behavior of fiber-reinforced restorations remains dependent on residual tooth structure, ferrule, post geometry, fiber orientation, cementation quality, adhesive interface, and occlusal loading. Recent systematic reviews indicate that the evidence comparing teeth restored with and without posts remains heterogeneous, and the potential benefit of post placement appears to depend substantially on the clinical situation and amount of remaining tooth structure.

Although Ribbond, everStickPOST, and Interlig are all classified broadly as fiber-reinforced restorative systems, they differ considerably in fiber composition, architecture, matrix characteristics, and handling behavior. These differences are clinically relevant because the architecture and orientation of reinforcing fibers influence stress transfer, crack propagation, adaptability, and integration with the resin restorative complex.

Ribbond is an UHMWPE fiber ribbon characterized by a woven, multidirectional architecture. Its surface treatment is intended to improve wettability and interaction with resin-based materials, thereby facilitating incorporation of the fibers within the surrounding resin matrix [12]. The woven architecture allows reinforcement in multiple directions and may contribute to redistribution of stresses and restriction of crack propagation within the composite restorative complex. This characteristic may be particularly advantageous when reinforcement is required across a relatively broad coronal defect or when adaptation to a nonuniform restorative geometry is necessary.

Long-term clinical evidence also supports the potential utility of fiber posts in appropriately selected endodontically treated teeth, with an overall tooth survival rate of 89.6% after a mean observation period of 8.8 ± 2.3 years in a long-term clinical cohort. The survival rate was 94.3% for teeth restored with fiber posts compared with 76.3% for teeth restored without posts [13]. However, these findings should be interpreted cautiously because the study was observational, and post-placement was determined according to the residual tooth structure and clinical treatment requirements rather than random allocation. Therefore, the higher survival observed in the fiber-post group should not be interpreted as definitive evidence of a causal effect of post placement.

everStickPOST differs from Ribbond in both fiber composition and architecture. It is an individually formable glass-fiber root canal post composed of unidirectional E-glass fibers embedded within a polymer matrix incorporating a semi-interpenetrating polymer network. Its adaptable configuration permits individualized shaping before final polymerization and can facilitate close adaptation to irregular or noncircular canal anatomy. This characteristic is particularly relevant when preservation of radicular dentin is prioritized, and a customized intracanal reinforcement is required.

The mechanical performance of glass-fiber systems is influenced by fiber composition, orientation, fiber volume, fiber-matrix interaction, and the efficiency of stress transfer between the reinforcing fibers and surrounding resin matrix. Surface treatment and the interaction between the fiber and resin matrix are also important because an effective fiber-resin interface facilitates transfer of functional stresses from the restorative material to the reinforcing fibers. Thus, the clinical advantage of an individually adaptable glass-fiber system should not be attributed solely to its material composition but also to its ability to conform to the anatomy of the prepared canal.

Comparative evidence between everStickPOST-type glass fiber systems and polyethylene fiber systems remains predominantly laboratory-based. A recent systematic review and meta-analysis of in vitro studies comparing Ribbond with other post systems found that Ribbond demonstrated lower fracture strength than several prefabricated metal and fiber posts but exhibited a more favorable pattern of non-catastrophic failure [14]. These findings suggest that fracture strength alone should not be considered the sole determinant of clinical performance. Importantly, because this evidence was derived from in vitro studies, it cannot be directly extrapolated to long-term clinical survival or clinical superiority among fiber systems.

Interlig represents another structural configuration, consisting of a braided glass-fiber reinforcement that can be incorporated into resin-based restorative materials. Its braided architecture permits adaptation to the contours of the restorative substrate and provides fiber reinforcement within the composite mass. In the present case series, Interlig was used in Case 3 as an intracanal reinforcement incorporated into a direct composite restoration. The clinical rationale was to reinforce the residual tooth structure while maintaining a conservative preparation and avoiding unnecessary extension of the restoration into a full-coverage prosthetic design.

The structural and handling differences among Ribbond, everStickPOST, and Interlig indicate that these systems should not be regarded as mechanically or clinically interchangeable. Ribbond provides multidirectional reinforcement through its woven polyethylene architecture; everStickPOST permits individualized adaptation of unidirectional glass fibers within the root canal; and Interlig provides braided glass-fiber reinforcement that can be incorporated into resin composite. Accordingly, the choice of fiber system should be individualized according to the quantity and geometry of residual tooth structure, canal morphology, extent of structural loss, required reinforcement, accessibility, adhesive strategy, and planned definitive restoration. The present three cases demonstrate different applications of these systems but do not provide sufficient evidence to establish the superiority of one system over another.

Bioactive glass has attracted considerable interest in restorative dentistry because its interaction with aqueous environments can promote ionic exchange and subsequent formation of a calcium- and phosphate-rich apatite-like mineral phase on treated surfaces [15]. Experimental evidence indicates that bioactive glass can promote apatite formation on dentin; however, the extent to which this translates into clinically meaningful remineralization and improvement in dentin mechanical properties remains uncertain. A systematic review found evidence of enhanced apatite formation following bioactive-glass treatment but emphasized the limited evidence regarding restoration of the mechanical properties of treated dentin.

In the present case series, bioactive glass was used only in Case 3 as an adjunctive dentin-biomodification procedure before intracanal fiber placement. The purpose was to introduce an additional biomodification step within the conservative restorative protocol. However, no quantitative assessment of mineral deposition, apatite formation, dentinal tubule occlusion, mineral content, or mechanical properties was performed. Therefore, the present case cannot establish that bioactive glass produced clinically meaningful remineralization or mechanical reinforcement. Its use should instead be regarded as an adjunctive and exploratory component of the restorative protocol. This distinction is important because much of the available evidence regarding bioactive glass and dentin remains laboratory-based, with limited clinical evidence supporting specific intracanal biomodification protocols.

Riboflavin combined with 0.2% chitosan was also used exclusively in Case 3 as an additional dentin pretreatment before fiber placement. Dentin biomodification strategies have been investigated with the objective of modifying the biochemical and biomechanical properties of dentin and improving the interaction between dentin and restorative materials. However, the clinical significance of the specific riboflavin-chitosan protocol used in the present case remains uncertain. As the present case series did not include a control condition or quantitative evaluation of dentin mechanical properties, collagen cross-linking, bond strength, mineral deposition, or long-term interface stability, no conclusion regarding therapeutic superiority can be drawn. The riboflavin-chitosan protocol should therefore be regarded as an investigational adjunct rather than an established clinical treatment.

An important distinction among the three cases was the choice of definitive coronal restoration. The decision to provide a full-coverage crown in Cases 1 and 2, while restoring Case 3 using direct composite restoration, was based on the amount and quality of remaining tooth structure and the anticipated biomechanical requirements of each tooth rather than on the fiber system itself. This distinction is clinically important because intracanal fiber reinforcement does not inherently determine whether a tooth requires full-coverage restoration. Instead, the definitive restorative design should be determined by residual axial walls, ferrules, pericervical dentin, functional loading, occlusal conditions, periodontal support, restorability, and the patient's individual requirements.

The ferrule remains an important consideration in the restoration of endodontically treated teeth. Evidence from systematic reviews indicates that the presence and extent of a ferrule can influence fracture resistance and, in some clinical analyses, restorative success. However, the magnitude of this effect varies according to ferrule height, circumferential continuity, residual wall configuration, dentin thickness, tooth type, and study design [16]. In Case 1, the extensive loss of coronal tooth structure and the resulting restorative requirements supported the use of a definitive full-coverage crown following fiber-reinforced core reconstruction. The decision was therefore based on the structural condition of the tooth rather than on the presence of the fiber post alone.

Conversely, direct composite restoration may represent a conservative definitive option when adequate sound tooth structure remains, and the restoration can be adequately supported by the residual axial walls and pericervical dentin. In Cases 2 and 3, the decision to avoid full-coverage crowns was based on the clinically favorable amount of remaining coronal and pericervical tooth structure and the feasibility of achieving adhesive restoration using direct composite resin. This approach also avoids the additional circumferential tooth reduction associated with conventional crown preparation.

Clinical evidence supports the feasibility of direct composite rehabilitation in appropriately selected endodontically treated teeth. In a three-year clinical study of endodontically treated premolars with limited structural loss, teeth restored with fiber posts and direct composite restorations were compared with teeth receiving fiber posts, direct composite build-up, and subsequent full-coverage metal-ceramic crowns [17]. The findings demonstrated that direct composite restoration represented a viable option in appropriately selected teeth. However, these results should not be generalized to teeth with extensive structural loss, compromised ferrule, unfavorable occlusal conditions, or substantially different tooth morphology.

The difference in restorative strategy among the three cases also highlights the importance of individualized treatment planning. Case 1 involved extensive coronal structural loss and therefore received a full-coverage crown after fiber-reinforced core reconstruction. In contrast, Cases 2 and 3 retained sufficient coronal and pericervical tooth structure to permit direct composite restoration. Thus, the use of an intracanal fiber does not, by itself, dictate the need for extracoronal coverage. Rather, the fiber should be regarded as one component of a comprehensive restorative strategy designed around the remaining tooth structure and the functional requirements of the tooth.

The clinical relevance of this approach is particularly apparent in anterior teeth, where preservation of tooth structure, esthetics, function, and repairability are important considerations. The use of different fiber architectures allows the reinforcement strategy to be adapted to the geometry and extent of the structural defect. In the present series, Ribbond, everStickPOST, and Interlig were used according to the individual restorative requirements of the treated teeth, demonstrating different approaches to conservative fiber reinforcement.

Importantly, these cases should be interpreted as clinical demonstrations rather than comparative evidence of superiority among the three fiber systems. The limited number of cases, absence of a control group, lack of standardized quantitative biomechanical assessment, and limited ability to isolate the effects of fiber architecture from the effects of residual tooth structure and definitive restoration preclude conclusions regarding the relative clinical effectiveness of Ribbond, everStickPOST, and Interlig. Similarly, the adjunctive bioactive glass and riboflavin-chitosan procedures used in Case 3 should be considered exploratory. Longer-term prospective clinical studies with standardized assessment of residual tooth structure, ferrule, fracture patterns, restoration survival, periodontal status, and periapical outcomes are required to determine whether these individualized fiber-reinforced approaches provide clinically meaningful advantages.

## Conclusions

Within the limitations of this three-case series, everStickPOST, Ribbond, and Interlig fiber-reinforced systems provided clinically satisfactory esthetic and functional rehabilitation of extensively compromised endodontically treated anterior teeth. The described approach emphasizes preservation of residual radicular and pericervical dentin while allowing individualized adaptation of intracanal fiber reinforcement to the remaining tooth anatomy. In the present cases, full-coverage restoration was used when extensive coronal structural loss compromised the ability of the remaining tooth to withstand functional loading, whereas direct composite restoration was used when sufficient sound tooth structure remained. Full-coverage restoration may be appropriate when extensive coronal structural loss compromises the ability of the remaining tooth to withstand functional loading, whereas direct composite restoration may be considered when sufficient sound tooth structure remains. Bioactive glass and riboflavin-chitosan pretreatment may have potential as adjunctive dentin-biomodification strategies; however, the present case series cannot establish their effectiveness. Larger prospective clinical studies with standardized protocols and long-term follow-up are required to determine the clinical longevity of fiber-reinforced intracanal reinforcement and to establish the role of adjunctive dentin biomodification.
